# A Race against the Clock: A Case Report and Literature Review Concerning the Importance of ADAMTS13 Testing in Diagnosis and Management of Thrombotic Thrombocytopenic Purpura during Pregnancy

**DOI:** 10.3390/diagnostics12071559

**Published:** 2022-06-27

**Authors:** Melinda Ildiko Mitranovici, Lucian Pușcașiu, Ioan Emilian Oală, Izabella Petre, Marius Lucian Craina, Antonia Rebeka Mager, Kinga Vasile, Diana Maria Chiorean, Adrian-Horațiu Sabău, Sabin Gligore Turdean, Ovidiu Simion Cotoi

**Affiliations:** 1 Department of Obstetrics and Gynecology, Emergency County Hospital Hunedoara, 14 Victoriei Street, 331057 Hunedoara, Romania; oalaioanemilian@gmail.com; 2 Department of Obstetrics and Gynecology, County Emergency Hospital, University of Medicine and Pharmacy Targu Mures, 38 Gh. Marinescu Str., 540142 Targu Mures, Romania; puscasiu@gmail.com; 3 Department of Obstetrics and Gynecology, Victor Babeș University of Medicine and Pharmacy, 2 Eftimie Murgu Sq., 300041 Timisoara, Romania; petre.izabella@umft.ro (I.P.); mariuscraina@hotmail.com (M.L.C.); 4 Department of Pathology, Emergency County Hospital Hunedoara, 14 Victoriei Street, 331057 Hunedoara, Romania; rebeka.dicu@yahoo.ro; 5 Department of Hematology, Emergency County Hospital Deva, 1 Decembrie Street, 330005 Deva, Romania; vasilekinga@yahoo.com; 6 Department of Pathology, County Clinical Hospital of Targu Mures, 540072 Targu Mures, Romania; chioreandianamaria@yahoo.com (D.M.C.); adrian-horatiu.sabau@umfst.ro (A.-H.S.); sabiturdean@yahoo.com (S.G.T.); ovidiucotoi@yahoo.com (O.S.C.); 7 Department of Pathophysiology, George Emil Palade University of Medicine, Pharmacy, Science and Technology of Targu Mures, 38 Gheorghe Marinescu Street, 540142 Targu Mures, Romania

**Keywords:** thrombotic thrombocytopenic purpura, von Willebrand factor protease, ADAMTS13, vascular microthrombosis, pregnancy, fetal–maternal mortality, placenta

## Abstract

Thrombocytopenic purpura (TTP) is a rare, potentially fatal pathology characterized by microangiopathic thrombotic syndrome and caused by an acute protease deficiency of von Willebrand factor, ADAMTS13. Moreover, ADAMTS13 deficiency promotes microthrombosis led by the persistence of ultra-large VWF multimers in the blood circulation. According to the few studies involving pregnant participants, the heterogeneity of manifestations has made this pathology difficult to diagnose, with an unexpected occurrence and increased risk of maternal and fetal morbidity and mortality. We reported on the case of a 28-year-old pregnant woman with an obstetric score of G2P0 who presented to the obstetrics and gynecology department of our clinic with the complaint of minimal vaginal bleeding. The evolution of our case was severe and life-threatening, a “race against the clock”, with our goal being to emphasize the importance and difficulty of diagnosing TTP in the absence of specific symptomatology. We faced a lack of technological support for a correct and complete diagnosis, and the first manifestation of this disease was the intrauterine death of the fetus. After completing all the necessary procedures, the placental tissue was sent for further histopathological evaluation. We highlighted the importance of monitoring ADAMTS13 for relapses monthly, with prophylaxis being essential for maternal and fetal mortality and morbidity.

## 1. Introduction

Thrombocytopenic purpura (TTP) is a rare and very aggressive pathology which endangers the patient’s life and is characterized by thrombocytopenia and hemolytic anemia with the clinical consequences of thrombosis; it is caused by ADAMTS13 (a disintegrin and metalloproteinase with a thrombospondin type 1 motif, member 13) deficiency.

The pathophysiologic mechanism consists of either protease deficiency due to the mutation of the ADAMTS13 gene (Upshaw–Shulman syndrome) or the development of specific inhibitory autoantibodies [1,2,3,4,5]. As it is fatal if not properly diagnosed and treated, TTP is three times more common in females, and half of those affected are pregnant or postpartum.

Dr. Eli Moschcowitz described this disease for the first time in 1924 in a 16-year-old female whose autopsy revealed disseminated thrombi in the terminal arterioles and capillaries of various organs. Later, in 1947, Singer named thrombotic thrombocytopenic purpura (TTP) and characterized it as a disease with a high-mortality rate (90%). The annual incidence rate, depending on demographic factors, occurs with a frequency of 1.5:1 million adults/year [6,7].

In terms of manifestations of the disease, it starts as an acute microangiopathy (TMA), characterized by microangiopathic hemolytic anemia, severe thrombocytopenia, and vascular microthrombi, caused by a severe deficiency of the von Willebrand factor cleavage protease ADAMTS13, a critically important enzyme that is synthesized in hepatic stellate cells [6,8,9,10].

As a result, the ischemic lesions that occur can affect any organ, including the placenta [11]. Acquired TTP with autoantibodies and severe ADAMTS13 deficiency (<10%) is the most common form of this disease [12,13,14].

The general risk factors for thrombotic thrombocytopenic purpura (TTP) include sepsis, neoplasia, the use of antiaggregant medication, organ transplantation, and pregnancy; otherwise, it is idiopathic. It can occur suddenly during pregnancy, the pregnancy state being the next high-risk period that can precipitate the appearance of a TTP, with an accentuated risk of morbidity and maternal–fetal mortality. It is more common in nulliparous patients whose estradiol levels are significantly higher than in multiparous patients [6,8,12]. Immunological changes during pregnancy are equally important. Pregnancy itself, as well as the postnatal period, represents a physiological procoagulant state and also a risk factor for accelerating the appearance of acute TTP. During pregnancy, changes in hemostasis and immunology predispose the patient to hypercoagulation that gradually returns to normal in approximately six-weeks postpartum. The concentration of von Willebrand factor (VWF) increases significantly in the third trimester of pregnancy, whereas ADAMTS13 decreases progressively starting with the second trimester, returning to its normal values on day 21 postpartum. In addition, estrogen control over the protease has a special role, as well [8].

There is an immunological tolerance to the semi-allogeneic fetus; this status favors the appearance of autoimmune pathologies, either de novo, such as acquired autoimmune disease by FVIII inhibitor, or relapses, such as recurrent lupus erythematosus [8,15]. On the contrary, several autoimmune diseases can occur simultaneously. Additional research could evaluate the types of autoantibodies highlighted in [8,15,16] for subsequent management.

Currently, the therapeutic decision may be delayed by the determination of ADAMTS13, which may lead to delays with serious consequences for the patient [6,17]. A first-line therapy based on clinical and paraclinical data has been initiated, consisting of plasmapheresis and the administration of fresh or frozen plasma [1,5,18]. Immunosuppression has been the cornerstone of treatment by administering steroids or targeting anti-ADAMTS13 antibodies with rituximab, as an initial therapy [6,17,19].

Caplacizumab, a humanized immunoglobulin derived from llamas, has the effect of inhibiting the interaction of VWF with platelets; it was the first drug specifically approved for the treatment of TTP and has been used as an initial therapy when available and also blocks organ damage caused by TTP. There has been ample evidence indicating that caplacizumab significantly reduced the time for the normalization of platelet counts, reducing the risk of exacerbation of the paraclinical picture [6].

Furthermore, a new agent such as ADAMTS13 recombinant is currently being investigated, and cyclosporine has been considered for treatment-resistant cases [6,20].

In 2013, Shortt et al. described the first case of refractory TTP treated with a proteasome inhibitor that targets plasma cells, bortezomib. The patient had undetectable ADAMTS13, the presence of inhibitory autoantibodies, and severe neurological symptoms [6,21].

In terms of other treatment, splenectomy has been considered, and it is a possible option for patients with chronic recurrence. It is usually effective, with a non-response rate of up to 8% in some reports and a survival rate of 70% at 10 years. Although, previously, splenectomy had an increased risk of adverse events, especially when used in refractory TTP, improvements in surgical techniques have significantly reduced complications, especially when using a laparoscopic technique [6,22].

N-acetylcysteine, an FDA-approved, anti-mucolytic agent, is a possible new treatment strategy for TTP, as it has been shown to reduce disulfide bonds in VWF, thereby reducing the size of VWF multimers and their prothrombotic potential. Studies in mice have shown that the prophylactic administration of N-acetylcysteine has been effective in preventing severe signs of TTP. This in vivo finding was supported by in vitro data, demonstrating the reduction properties of VWF multimers. In a group of baboons, however, N-acetylcysteine administration did not eradicate the paraclinical signs of pre-existing TTP; thrombocytopenia, hemolytic anemia, and organ damage could not be restored to normal. However, a reduction in VWF multimers was observed, demonstrating the efficacy of N-acetylcysteine in reducing disulfide bonds in circulating VWF multimers. N-acetylcysteine appeared to inhibit VWF-dependent platelet aggregation, but has currently only been studied in animals. Differential diagnosis with other TMAs (acute microangiopathies) has been very difficult to establish [6,22,23]. The relapse rate of this pathology recorded in studies is still relatively high with long-term follow-up and subsequent management being required along with multidisciplinary-instituted therapy [17,18,24,25].

## 2. Case Report

A 28-year-old pregnant woman at 27 weeks gestation (gravida 2 para 0) presented on the 29 March 2021 in the obstetrics and gynecology department of our hospital with minimal vaginal bleeding, normal viable fetus, lateral placenta previa, and uterine myoma. From the past medical history of the patient, we noted a miscarriage in the second trimester of pregnancy.

General information about the patient was as follows: 67 kg weight, 165 cm height; she had urban citizenship; she graduated from secondary school, and her socioeconomic status was normal. The patient benefited from antianemic treatment and vitamins, and she followed a specific diet for pregnancy; she was a nonsmoker and did not have any comorbidities.

At admission, the laboratory tests showed pregnancy anemia: hemoglobin (Hgb), 10.9 g/dL (normal range 11.5–16); hematocrit (Htc), 34.8%; the number of platelets, 143 × 109/L (normal range 150 × 109/L–450 × 109/L); the number of leukocytes, 11.3 × 109/L (4 × 109/L–10 × 109/L); PCR, 1.93 mg/L (normal); no bacterial growth in cultures; normal U and E; and normal liver and renal tests. Symptomatology had improved with antispasmodic and antianemia treatment.

She returned to our department on the 19 April 2021 at 17:25 with vaginal bleeding and IUFD. The clinical picture of the case consisted of vaginal bleeding, absent fetal movements without other symptoms, blood pressure (BP) at 100/70 mmHg, and a heart rate (HR) of 72 beats per minute. Admission laboratory tests revealed severe anemia: hemoglobin (Hgb), 5.5 g/dL; hematocrit (Htc), 17.4%; leukocytes, 16.2 × 109/L; and severe thrombocytopenia with platelets, 11 × 109/L. Therefore, the intensive care unit doctor was contacted. Biochemistry showed that there were small changes in urea level, 50.47 mg/dL; creatinine level was 1.38 mg/dL; uric acid, 6.947 mg/dL; GOT, 91 U/L; and PCR, 79.04 mg/L; the coagulation samples were normal. At 21:00, she spontaneously delivered a 1300 g, lifeless female fetus, with no delivery complications or postpartum hemorrhage. Oxytocin was administered during placental delivery. Obstetric maneuvers to reduce the risk of bleeding were not required, and she was transferred to the intensive care unit (ICU).

While in the ICU, the patient benefited from a specific treatment: red blood cell (RBC), platelet (PLT), and fresh frozen plasma (FFP) transfusion, dexamethasone, antibiotics (meropenem, metronidazole), dalteparin sodium 5000 IU, and prophylactic uterotonics.

On the first day after birth, supplementary tests were required to elucidate the diagnosis and follow up. The peripheral blood film showed reticulocytosis, 66/1000 RBC (normal 5–15/1000 RBC); anisochromia and hypochromia; anisocytosis; mild microcytosis; and frequent target erythrocytes (aspect that was consistent), especially schizocytes, which repeatedly raised our suspicion of TTP.

Other blood tests showed normal levels of direct bilirubin; GOT. 94 U/L; serum creatinine, 1.54 mg/dL (without increase); urea, 51.03 mg/dL; coagulation blood tests INR, 1.09; aPTT, 23.5 s; fibrinogen, discreetly increased to 453 mg/dL (normal being up to 450 mg/dL); total protein 5.62 g/dL; blood type OI, Rh positive; hemoglobin (Hgb), 6.9 g/dL; hematocrit (Htc), 23.0%; leukocytes, 14.2 × 109/L; and platelets, 18 × 109/L. We concluded that anemia and thrombocytopenia remained low, despite RBC and platelet (PLT) transfusion. The cervical swab culture and the uroculture did not detect bacterial colonization. When red cell concentrate (RCC) transfusion was administered, our patient developed pulmonary edema (ischemic emboli), which was subsided by specific treatment, oxygen and pulmonary taping and anticoagulant. Hematological consult was required through telemedicine, due to the suspicion of TTP, which confirmed the diagnosis and guided us to the transfer solution in a center included in the National Program of Acquired Thrombocytopenic Thrombotic Purpura. After completing these steps, we continued the investigations to determine the risks of thrombosis: D-dimers were determined with a result of >3000 ng/mL (normally below 100 ng/mL in our laboratory). On the 21 April 2021, the troponin level was 7.11 ng/mL (normally below 0.5 ng/mL), with an increased risk of ischemia.

Considering the evolution of the patient; the presented determinations; the normal BP; the blood tests—especially the anemia with schizocytes on peripheral smear and the severe thrombocytopenia; the uncorrectable with PLT transfusion; the increased levels of LDH from 1374.8 U/L to 1764.6 U/L; the troponin and D-dimer levels; slightly increased fibrinogen; and normal aPTT, the evidence supported our suspicion of TTP. Moreover, at the point we could exclude obstetric pathology, we were advised and guided for further management of the case by a hematologist from Deva County Hospital, due to the lack of a hematology service in our clinic at that time.

For a correct differential diagnosis, we divided the patient’s tests as follows (Table 1 and Table 2):

During this time, a transfer to a hospital with appropriate facilities was being sought, especially as the recommended plasmapheresis could not be performed without hematological supervision. Even though PLT administration has caused controversy due to the microthrombi it can generate [9,26], Swisher showed it to be useful in very low hemoglobin levels and severe thrombocytopenia, due to high risks of bleeding [26].

**Figure 1:**
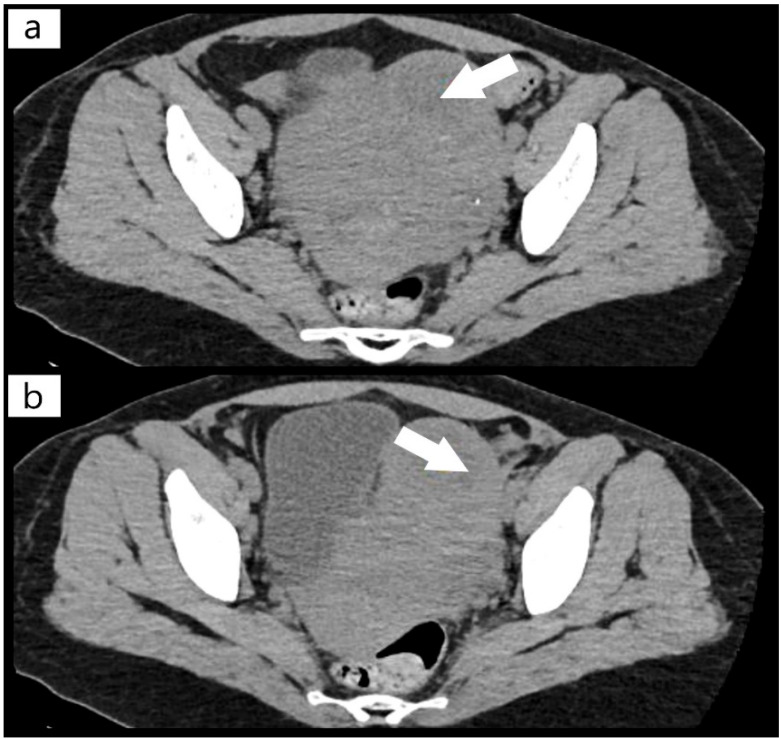
(**a**,**b**) Computer tomography (CT) scan with fibroid mass of the uterus (arrows).

**Figure 2:**
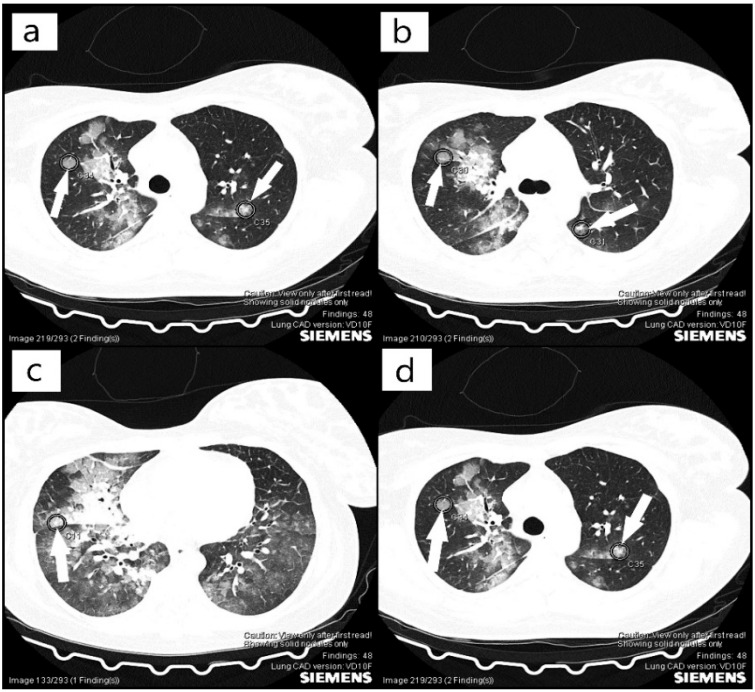
(**a**–**d**) Computer tomography (CT) scan with pulmonary microthrombosis (arrows).

On the 22 April 2021, we obtained the patient’s transfer to the intensive care unit of Hematology Sibiu, a hospital accredited for the treatment of this pathology through the Acquired Thrombocytopenic Thrombotic Purpura Program, part of the National Program of Rare Genetic Diseases. During this time, a CT (computer scanner) scan was required, which highlighted the fibromatous uterus with a 6 cm fibroid mass (Figure 1) and pulmonary edema, with pulmonary microthrombosis (Figure 2). Her cardiac examination revealed slight ischemic changes. Until her transfer, the only symptoms of the patient were mild fatigue and blurred vision, but no bleeding, neurological symptoms, or renal manifestations, which, once again, emphasized the heterogeneity of this disease that makes it difficult for a clinician to diagnose. In the literature, the diagnosis of TTP is still based on the clinical picture and laboratory tests, and the treatment should be initiated as quickly as possible before determining ADAMTS13, as TTP underdiagnosed and undertreated can quickly turn lethal [11,13,14]. The patient’s relatives were instructed to request a specific, semi-quantitative ADAMTS13 analysis, which was subsequently performed, for which we would like to thank the representative of the National Program for Rare Genetic Diseases, who supported our efforts for a definite diagnosis.

In this case, the pathology appeared de novo, suddenly and brutally, and the trigger factor was pregnancy.

The potential mechanism of this pathology consists of elevated levels of VWF multimers causing increased oxidative stress. This results in the appearance of thrombi in the small vessels. In our patient’s case, the mechanism was the development of specific inhibitory autoantibodies. This microangiopathy led to erythrocyte fragmentation, resulting in the formation of schizocytes. The underlying pathologic mechanism was thrombosis, causing neurological and renal failures. The development of thrombi and the microangiopathy led to hemolytic anemia and thrombocytopenia. In our case, the infarction process was apparent in the placental tissue, heart, and lungs, which was more unusual.

**Figure 3:**
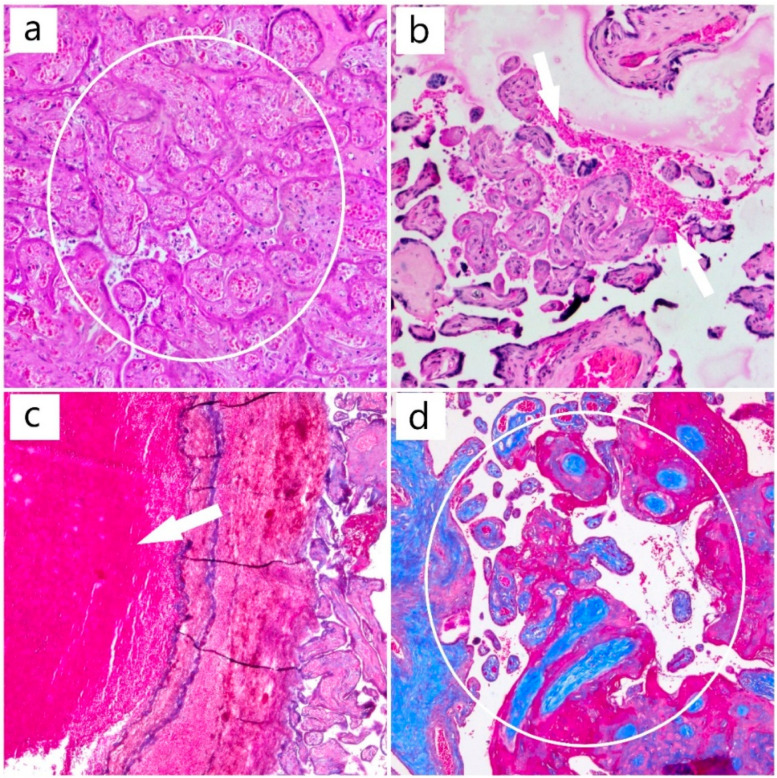
(**a**) Area of placental infarction (HE, ob. 10×) (circle); (**b**) areas of intervillous hemorrhage (HE, ob. 10×) (arrow); (**c**) mixed thrombus (HE, ob. 10×) (arrow); (**d**) chorionic villi (Masson’s trichrome special stain, ob. 10×) (circle).

The histopathology of the placenta, on routine histological stain (hematoxylin and eosin), revealed the presence of circulatory disorders such as intravenous thrombosis and ischemic villous necrosis, as well as dystrophic changes with fibrinoid deposits and areas of intervillous hemorrhage (Figure 3).

**Figure 4:**
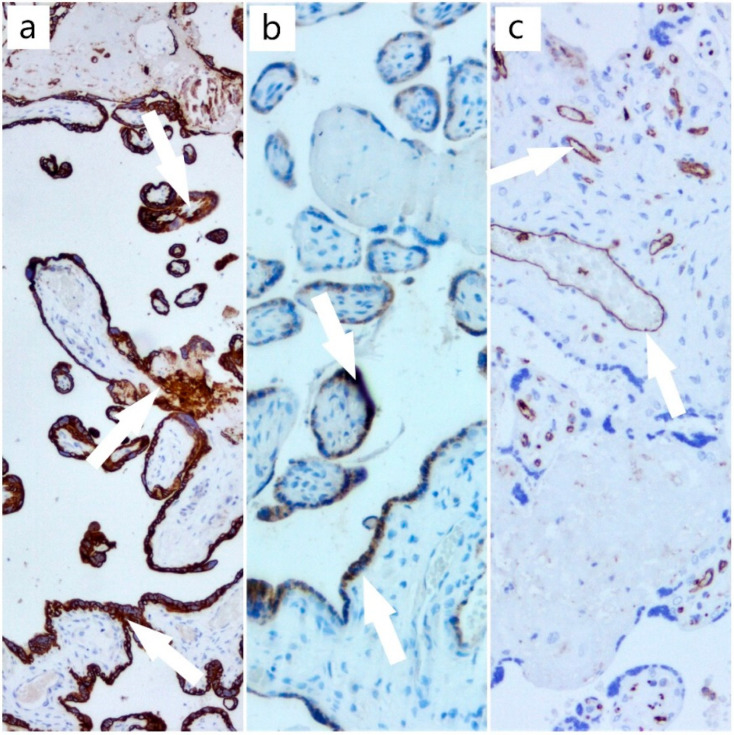
(**a**) CTK AE1/AE3 immunostain, ob. 5× (positive trophoblast with arrow); (**b**) β-hCG immunostain, ob. 10× (positive trophoblast with arrow); (**c**) CD31 immunostain, ob. 10× (positive for vascular endothelium from axis of chorionic villi with arrow).

The results of histochemical and immunohistochemical techniques using Masson’s trichrome stain and the antibodies against CTK AE1/AE3, β-hCG, and CD31 provided data regarding the aggressiveness of this disease (Figure 4), with multiple infarctions that endangered the health of the fetus, but also the mother, with the possibility of complications, such as preeclampsia and hemolysis, elevated liver enzymes, and low PLTs (HELLP) syndrome. In our case, the fetal death was due to induced hypoxia.

While in the ICU, the patient benefited from a specific treatment: red blood cell (RBC), platelet (PLT), and fresh frozen plasma (FFP) transfusion, dexamethasone, antibiotics (meropenem, metronidazole), dalteparin sodium 5000 IU, and prophylactic uterotonics. A transfer to a hospital with appropriate facilities was being sought, for specific hematological treatment.

In the meantime, we were given the result for ADAMTS13 which was 1.5% (<10%).

After the patient was transferred to a tertiary referral hospital, she immediately responded to the treatment with caplacizumab and plasma exchange. To date, the patient’s status is good. The National Program for Rare Genetic Diseases is a national organization that provides funds for rare diseases as well as to increase awareness of the program among the national maternity units. Our patient was the first included in this program and treated according to the new protocol.

## 3. Discussion

Pregnancy is a factor that can precipitate the appearance of TTP, with the potential for relapse in following pregnancies. Few studies have focused on this issue, and the heterogeneity of disease manifestations have made it difficult to diagnose and manage [10,12,17,20]. The physiological and immunological changes during pregnancy relate to the unique manifestations of TTP during this period that have yet to be fully understood through research. A multidisciplinary team composed of an obstetrician, a hematologist, an anesthesiologist, a laboratory doctor, and a neonatologist is mandatory for an accurate diagnosis.

In the present case, the first manifestation of the illness was the intrauterine death of the fetus, at 28 weeks gestation. The evolution of this case was life-threatening and created “a race against the clock”. In addition, we were confronted with a lack of technological support for a correct and complete diagnosis: the absence of a hematologist for the use of plasmapheresis as a treatment in accordance with clinical guidelines as well as the absence of an ADAMTS13 diagnostic kit, which should be available in all hospitals.

A prompt diagnosis can save a patient’s life. For example, in [10], the diagnostic error led to the patient’s death [10]. The patient was a 30-year-old primigravida, at week 28 of pregnancy. Her symptoms were dizziness, diplopia, petechia, and headache; then lung oedema developed as well as, a few days later, cardiopulmonary arrest. The doctors decided on a perimortem caesarean section. A few hours later, the doctors declared her death, though the newborn survived [10]. We can easily encounter such situations in our practice, due to the rarity of the disease and the difficulty of recognizing it. Thus, this case highlights the ease of misdiagnosis and its possible future consequences.

Our patient’s symptoms were different: mild fatigue, blurred vision, no bleeding or neurological symptoms, and no petechia, which once again emphasizes the heterogeneity of this disease. However, in all cases, the evolution is severe.

Including our patient in the National TTP Program was essential, as she was the first patient who benefitted from the initiation of the caplacizumab treatment in Romania. Maternal–fetal complications (IUGR, premature birth, fetal death, maternal stroke) have been common and can be difficult for the patient [14,27], and the relapse rate is 100% [8,11,18,23,24]. It has been postulated that proteins found in the placental circulation may serve as maternal triggers for the development of anti-ADAMTS13 autoantibodies and may be associated with pregnancy complications [13,14,26,27]. Histopathological analysis of the placenta can provide valuable data on how the vascular thrombosis and the characteristic microangiopathy of TTP occurs. ADAMTS13 monitoring levels should be supported to prevent vascular microthrombosis that may affect the placenta. For a subsequent pregnancy, a period of 12 months from the last treatment has been indicated, and ADAMTS13 must be >25% prior to conception [10,24].

In future management of the disease in relation to pregnancy, especially since the results can be dire in spite of treatment, we emphasize the importance of administering the ADAMTS13 blood test to determine a correct diagnosis. All gynecology and obstetrics departments, at any level, should have access to this type of test.

In addition, following the blood tests, troponin and D-dimer tests should be included for a proper evaluation of ischemia in TTP. The correct treatment for TTP associated with pregnancy should include plasma exchanges, corticosteroid therapy, and low molecular weight heparin (LMWH) treatment, to prevent thrombosis. If hemorrhage is a risk, a PLT transfusion should be used.

Moreover, the treatment should be adjusted according to the ADAMTS13 levels, the patient’s response to the treatment, and careful obstetrical evaluation, which will require a multidisciplinary as well as a telemedicine integration. The role of the neonatologist is considered in the third trimester of pregnancy, for further evaluation of the newborn [28,29,30].

The incidence of a TTP affecting adults amounts to 2.55 million/year in Europe and 2.39 million/year worldwide; therefore, we can conclude that it is a very rare disease [31]. The prevalence, according to the latest cross-sectorial study using a meta-analysis was 13 cases/1 million people in Europe and 19/1 million people in the United States [31]. Romania does not have this statistical data available at present: our patient was the first to be included in the National Program for Acquired Thrombotic Thrombocytopenic Purpura and was following the latest medication protocol.

A simple, easy-to-understand reference with a rapid differential diagnostic algorithm would be useful. In addition, the management of subsequent pregnancies and monthly follow-up of ADAMTS13 for signs of relapse are critical. According to the literature, the frequency of relapse is extremely high [16,17,24,25]. In our study, the support therapy was decisive and the corticotherapy, LMWH anticoagulant therapy, and tests for TTP made the difference. Troponin and D-dimers, which were deeply altered in our case, were essential, and changes of their levels have not been taken into account in other studies for the evaluation of thromboembolism risk in TTP.

Even if there are no relapses in pregnancy, the risks of fetal mortality, fetal complications such as IUGR, miscarriage, and preeclampsia have persisted in the literature [8,13,27]. In addition, a new agent such as ADAMTS13 recombinant is currently being investigated, and cyclosporine has been considered for treatment-resistant cases [6,20].

At the present time, we are facing low-to-moderate certainty regarding the recommendations; there is not enough data to determine the most efficient therapeutic approach. Multidisciplinary guidelines are required, as are innovative treatments, although there have been limitations on high quality data regarding treatments [20].

## 4. Conclusions

The correct diagnosis through ADAMTS13 determination, as well as all the other investigations used in our patient’s case, were critical for the good outcome achieved. Obstetricians should be aware of this rare and fatal disease, and multidisciplinary approaches are highly recommended to reduce the risk of misdiagnosis.

Prophylaxis is essential. Monitoring the ADAMTS13 serum levels is necessary in planning a subsequent pregnancy, and the initiation of plasmapheresis prior to receiving the results of ADAMTS13 can be helpful.

Last but not least, we should consider the long-term complications of TTP in terms of stroke, hypertension, ischemic heart disease, cognitive impairment, decreased quality of life, and the high mortality rate. We emphasize that the use of anticoagulants is essential for survival.

Additional tests should be administered to support this diagnosis, including measuring the serum level of troponin and D-dimers along with LDH and the quantification of schizocytes from a peripheral blood smear.

Due to the rarity of this disease and the low number of studies on it, future research opportunities abound, especially in terms of associative and high-quality studies, as well as for therapeutic innovation.

## Figures and Tables

**Table 1:** Differential diagnosis.

Differential Diagnosis		
Diagnostic	Characteristics	
**TTP**	▪ Severe thrombocytopenia ▪ Severe microangiopathic anemia with reticulocytes ▪ Presence of schizocytes on peripheral blood smear (PBS) ▪ Increasing LDH levels and LDH/ASAT ratio > 10 ▪ Excessively high troponin levels ▪ Slightly elevated creatinine levels ▪ Elevated fibrinogen levels ▪ ADAMTS13 levels < 10%	
**DIC**	▪ Prolonged aPTT ▪ Low fibrinogen levels ▪ Severe thrombocytopenia (high D-Dimer levels) ▪ Presence of schizocytes on peripheral blood smear (PBS) ▪ Normal levels of ADAMTS13	
**Disseminated Lupus Erythematosus** **(Vasculitis from Antiphospholipid syndrome)**	▪ Increased intensity of PCR testing ▪ Occurrence of autoantibodies ▪ Moderate anemia ▪ Leukopenia ▪ Increased ESR ▪ Renal and hepatic impairment	
**HELLP Syndrome and Preeclampsia**	▪ High blood pressure ▪ High levels of LDH, but LDH/ASAT ratio < 10, so I delProteinuria ▪ Anemia ▪ Trombocytopenia when the fetus is delivered, the patient begins to recover, which is not our patient’s case.	

		not more than in PTT

**Hemolytic Uremic Syndrome**	▪ Creatinine level > 2 mg/dL ▪ Platelet count not as low as in TTP	
**Viral Infection** **Acute Sepsis** **COVID-19,** **Vaccination with Pfizer BioNTech (BNT2b2)**	▪ Leukocytosis or leukopenia ▪ Increased intensity of PCR testing ▪ Recent vaccination ▪ Coronavirus disease (COVID-19) was not present, at that time, in our country	
**Acute Fatty Liver**	▪ Digestive symptoms ▪ Upper abdominal pain ▪ Abnormal liver tests ▪ Leukocytosis ▪ Prolonged prothrombin and aPTT ▪ Hypofibrinogenemia ▪ Low platelet count ▪ Anemia	

**TTP**—thrombotic thrombocytopenic purpura, **DIC**—disseminated intravascular coagulation, **HELLP**—hemolysis, elevated liver enzymes and liver platelets, **aPTT**—activated partial thromboplastin time, **PCR**—polymerase chain reaction, **LDH**—lactate dehydrogenase, **ASAT**—aspartate aminotransferase.

**Table 2:** Blood tests results per day.

	Time of Admission								
	19 April 2021		20 April 2021		21 April 2021		22 April 2021		
Tests	Result 1	Result 2	Result 1	Result 2	Result 1	Result 2	Result 1	Result 2	Normal Value
**Leukocytes** **(WBC)**	14.7 × 10^9^/L	16.2 × 10^9^/L	14.2 × 10^9^/L	-	14.8 × 10^9^/L	17.4 × 10^9^/L	15.8 × 10^9^/L	-	4.00–10.00 × 10^9^/L
**Hemoglobin** **(HGB)**	5.3 g/dL	5.5 g/dL	6.9 g/dL	-	6.1 g/dL	8.0 g/dL	7.7 g/dL	-	11.5–16 g/dL
**Hematocrit** **(HCT)**	17.2%	17.4%	23%	-	20.7%	26.5%	25.5	-	35–48%
**Platelets** **(PLT)**	13 × 10^9^/L	11 × 10^9^/L	18 × 10^9^/L	-	22 × 10^9^/L	32 × 10^9^/L	26 × 10^9^/L		150–450 × 10^9^/L
**INR** **(International Normalized Ratio)**	1.05	-	1.09	-	1.1	-	1.04	-	0.8–1.2
**APTT**	29.1	-	25.1	-	25.5	-	26.5		25–38 s
**Fibrinogen**	410 mg/dL	-	453 mg/dL	-	387 mg/dL	-	415 mg/dL	-	180–450 mg/dL
**Creatinine**	1.36 mg/dL	1.38 mg/dL	1.54 mg/dL	-	1.48 mg/dL	1.62 mg/dL	1.56 mg/dL	-	0.50–0.90 mg/dL
**Urea**	53 mg/dL	50.47 mg/dL	51.03 mg/dL	-	60.06 mg/dL	58.86 mg/dL	69.96 mg/dL	-	16–43 mg/dL
**Uric Acid**	6.94 mg/dL	6.97 mg/dL	-	-	-	-	-	-	2.3–6.10 mg/dL
**Total Bilirubin**	-	-	1.30 mg/dL	-	1.66 mg/dL	-	1.39 mg/dL	-	0.30–1.10 mg/dL
**Direct Bilirubin**	-	-	0.37 mg/dL	-	0.31 mg/dL	-	0.49 mg/dL	-	0.10–0.40 mg/dL
**C Reactive Protein (CRP)**	75.5 mg/L	79.04 mg/L	85.47 mg/L	-			84.5 mg/L	-	0.00–5.00 mg/L
**Lactate dehydrogenase** **(LDH)**	-	-	-	-	1374 U/L	1582 U/L	1764 U/L	-	0.00–247.00 U/L
**Total Protein**	-	-	5.6 g/dL	-	5.26 g/dL	5.71 g/dL	5.84 g/dL	-	6.60–8.30 g/dL
**Peripheral Blood Smear**	Frequent target erythrocytes and schizocytes, aspect that is consistent								
**Reticulocytes**	Reticulocytes 66 per 1000 red blood cells are found (normal is 5–15 per 1000 red blood cells)								
**Troponin I**					7.11 ng/mL				<0.5 ng/mL
**D-dimers**					>3000 ng/mL				<100 ng/mL
**COVID19** **(PCR)**	Negative								
**Cultures**	All cultures were negative								
